# Impact of low-density lipoprotein cholesterol and lipoprotein(a) on mid-term clinical outcomes following coronary artery bypass grafting: A secondary analysis of the DACAB trial

**DOI:** 10.3389/fcvm.2023.1103681

**Published:** 2023-03-24

**Authors:** Qixiang Yu, Qing Xue, Hao Liu, Junlong Hu, Rui Wang, Yuanyuan Song, Yanzai Zhou, Wei Zhang, Yunpeng Zhu, Qiang Zhao

**Affiliations:** ^1^ Department of Cardiovascular Surgery, Ruijin Hospital, Shanghai Jiao Tong University School of Medicine, Shanghai, China; ^2^ Department of Cardiovascular Surgery, The First Affiliated Hospital of Naval Medical University, Shanghai Changhai Hospital, Shanghai, China; ^3^ Department of Cardiothoracic Surgery, Xinhua Hospital, Shanghai Jiao Tong University School of Medicine, Shanghai, China; ^4^ Department of Cardiac Surgery, Heart Center of Henan Provincial People’s Hospital, Central China Fuwai Hospital of Zhengzhou University, Zhengzhou, China; ^5^ Department of Cardiovascular Surgery, Nanjing First Hospital, Nanjing Medical University, Nanjing, China; ^6^ Department of Cardiovascular Surgery, Jiangsu Province Hospital, Nanjing, China; ^7^ Department of Biostatistics, School of Public Health, Fudan University, Shanghai, China

**Keywords:** coronary artery bypass grafting, lipoprotein(a) [Lp(a)], low-density lipoprotein cholesterol (LDL-C), lipids, major adverse cardiovascular events (MACE)

## Abstract

**Purpose:**

The objective was to evaluate the influence of low-density lipoprotein cholesterol (LDL-C) and lipoprotein(a) [Lp(a)] on clinical outcomes in patients undergoing coronary artery bypass grafting (CABG).

**Methods:**

This is a secondary analysis of a 5-year follow-up of the DACAB trial (NCT02201771), in which 500 patients who underwent primary isolated CABG were randomized to three-antiplatelet therapy for 1 year after surgery. Of them, 459 patients were recruited in this secondary analysis. Baseline LDL-C and Lp(a) levels were collected, and repeated measurement of LDL-C levels during the follow-up were recorded. Cut-off values for LDL-C were set at 1.8 and 2.6 mmol/L; thus, the patients were stratified into LDL-C <1.8, 1.8–<2.6, and ≥2.6 mmol/L subgroups. Cut-off value for Lp(a) was 30 mg/dL; thus, the patients were divided into Lp(a) <30 and ≥30 mg/dL subgroups. The primary outcome was 4-point major adverse cardiovascular events (MACE-4), a composite of all-cause death, myocardial infarction, stroke, and repeated revascularization. Median follow-up time was 5.2 (interquartile range, 4.2–6.1) years.

**Results:**

During the follow-up, 129 (28.1%) patients achieved the attainment of LDL-C <1.8 mmol/L, 186 (40.5%) achieved LDL-C 1.8–<2.6 mmol/L, and 144 (31.4%) remained LDL-C ≥2.6 mmol/L. Compared with the postoperative LDL-C <1.8 mmol/L group, the risk of MACE-4 was significantly higher in the LDL-C 1.8–<2.6 mmol/L group [adjusted hazard ratio (aHR) = 1.92, 95% CI, 1.12–3.29; *P* = 0.019] and LDL-C ≥2.6 mmol/L group (aHR = 3.90, 95% CI, 2.29–6.64; *P* < 0.001). Baseline Lp(a) ≥30 mg/dL was identified in 131 (28.5%) patients and was associated with an increased risk of MACE-4 (aHR = 1.52, 95% CI, 1.06–2.18; *P* = 0.022).

**Conclusions:**

For CABG patients, exposure to increased levels of postoperative LDL-C or baseline Lp(a) was associated with worse mid-term clinical outcomes. Our findings suggested the necessity of achieving LDL-C target and potential benefit of adding Lp(a) targeted lipid-lowering therapy in CABG population.

## Introduction

Coronary artery bypass grafting (CABG), the most commonly performed cardiac surgery, is considered to be the most effective revascularization strategy for several subsets of patients with coronary artery disease (CAD) (1). CABG has significantly improved the prognosis of patients with higher disease burden, complex coronary lesions and in the presence of diabetes, as compared to percutaneous coronary intervention (PCI) (2, 3). Even after surgical revascularization, these patients are stratified as a very high risk population susceptible to subsequent cardiovascular events, leading to mortality and morbidity. The effectiveness of CABG is affected by the progression of atherosclerosis occurred in native coronary arteries (4). In addition, accelerated atherosclerotic progression in the grafts usually compromises the success of CABG as well (4). Thus, lipid management remains the cornerstone strategy in secondary prevention after CABG (5).

Low-density lipoprotein cholesterol (LDL-C), the most fundamental and important factor in the development of atherosclerosis, is regarded as an extensively studied, well-established modifiable risk factor and the main target in the primary and secondary prevention of cardiovascular disease (CVD) (6). Elevated LDL-C is associated with increased cardiovascular events and event reduction has been proved to be proportional to the magnitude of LDL-C lowering (7). Thus, in patients with CAD, optimization of LDL-C and statin prescription is associated with reduced morbidity and mortality (6). For CABG patients, lowering postoperative LDL-C with statins and proprotein convertase subtilisin/kexin type 9 (PCSK9) inhibitors has been proved to be effective in reducing residual cardiovascular risk (7–9). Despite their effectiveness and clinical benefits, statins still remain underused and the achievement of LDL-C target is not satisfactory (10, 11).

Besides LDL-C, lipoprotein(a) [Lp(a)] is recently recognized as a long underestimated cardiovascular risk factor (12, 13). Lp(a) is causally and independently associated with increased risk of CVD (14). In secondary prevention settings, some studies proved that Lp(a) was associated with adverse cardiovascular events (15, 16). Results from a patient-level meta-analysis revealed that elevated baseline and on-statin treatment Lp(a) were independently related with cardiovascular disease risk (17). However, it remains inconclusive whether increased Lp(a) is associated with worse clinical outcomes in CABG patients.

The relationship between lipid profiles and clinical outcomes in patients following CABG is not well understood. Thus, we conducted a secondary analysis to investigate the impact of LDL-C and Lp(a) on mid-term major adverse cardiovascular events (MACE) in patients following CABG.

## Methods

### Study population

The DACAB trial was a prospective, multicenter, randomized trial. Briefly summarized, 500 patients from six Chinese tertiary hospitals were randomized to ticagrelor 90 mg twice daily plus aspirin 100 mg once daily, ticagrelor alone 90 mg twice daily, or aspirin alone 100 mg once daily for 1-year open-label antiplatelet therapy after CABG between July 2014 and November 2015. The primary outcome was 1-year vein graft patency at the graft level. The trial design and eligibility criteria have been published previously (18). In this secondary analysis, patients who had LDL-C and Lp(a) measured at baseline and LDL-C repeatedly measured during the follow-up period were pooled. Thus, 459 patients in the DACAB trial formed the present analysis. This study was approved by the independent institutional review board of Ruijin Hospital, Shanghai Jiao Tong University School of Medicine.

### Study procedures

Baseline LDL-C and Lp(a) were obtained when patients were admitted to the participating hospitals. Repeated measurements of LDL-C were required at 1, 3, 6, and every 12 months after CABG. The average value of these LDL-C levels during the follow-up was used to represent the postoperative LDL-C level. The patients were stratified into LDL-C <1.8, 1.8–<2.6, and ≥2.6 mmol/L subgroups. We adopted the sequential thresholds from the 2016 European Guidelines on cardiovascular prevention in consideration of the study period (19). Lp(a) was evaluated by an immunoturbidimetric method according to the manufacturer's instructions with a normal value of <30 mg/dL; thus, the patients were classified into Lp(a) <30 and ≥30 mg/dL subgroups. We did not repeat Lp(a) measurement for the reason that Lp(a) was considered genetically determined by LPA gene and the concentration remained relatively stable and was refractory to lifestyle and drugs including statins (20).

### Clinical outcomes and follow-up

The primary outcome was 4-point MACE, composed of all-cause death, myocardial infarction (MI), stroke, and repeated revascularization (MACE-4). Secondary outcomes included MACE-3 and MACE-5. MACE-3 was composed of cardiovascular death (CV death), MI, and stroke. MACE-5 was composed of all-cause death, MI, stroke, repeated revascularization, and rehospitalization for unstable angina. All-cause death included cardiovascular and non-cardiovascular death. CV death was referred to any death caused by cardiovascular cause and any death that had no identified reason. MI was composed of ST-segment elevation MI (STEMI) or non-STEMI (NSTEMI). Stroke was defined as new focal neurological deficit lasting >24 h and adjudicated by a neurologist based on image records. Patients were contacted at 1, 3, 6, and every 12 months after surgery. All end point events were adjudicated and reviewed by the independent adjudication committee. Antiplatelet therapy was prescribed according to the randomization for the first year, and then other secondary prevention medications were prescribed based on recommendation from the latest guidelines. Information about the use of secondary prevention medication was updated every 3 months during the first year after CABG.

### Statistical analysis

Continuous variables were expressed as mean with standard deviation (SD) or median with interquartile range (IQR). Categorical variables were presented as frequencies with percentages. Continuous variables were compared by using Student's *t*-test or one-way ANOVA and categorical variables were examined by using Fisher's exact test or Chi-squared test. The results of time-to-event analyses were carried out by using the Kaplan–Meier estimates, and the log-rank test was used to calculate the statistical difference. Cox proportional hazards model was used to estimate hazard ratios (HRs) with 95% confidence intervals (CIs). These models were adjusted for traditional cardiovascular risk factors and statistically significant variables in the subgroup comparisons. All analyses were performed by using SAS version 9.4 (SAS Institute Inc.). All statistical tests were two-sided and *P* values <0.05 were considered statistically significant.

## Results

Among the 500 patients, 41 were excluded due to incomplete lipid profiles. This study population had a mean age of 63.2 ± 8.1 years and 81.5% were male.

### Baseline LDL-C and clinical outcomes

**Figure 1:**
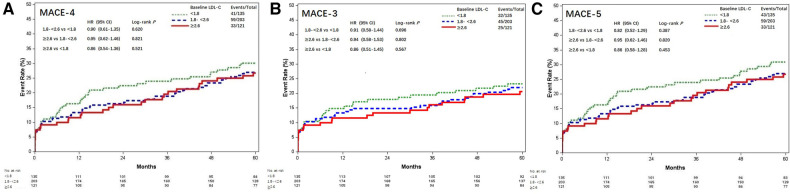
Kaplan–Meier curves of MACE among different baseline LDL-C levels (mmol/L). Kaplan–Meier estimates for freedom from (**A**) MACE-4, (**B**) MACE-3, and (**C**) MACE-5. MACE, major adverse cardiovascular events; LDL-C, low-density lipoprotein cholesterol; MACE-4, 4-point MACE; MACE-3, 3-point MACE; MACE-5, 5-point MACE.

These patients were stratified into three groups according to the baseline LDL-C levels. Clinical characteristics among the three groups were compared. Patients with increased baseline LDL-C were tended to be younger, more associated with on-pump CABG and less prescribed with angiotensin-converting enzyme inhibitor (ACEI) or angiotensin receptor blocker (ARB) at discharge. Increased baseline LDL-C levels were associated with lower prevalence of previous MI and hypertension (Table 1). There was no loss to follow-up. MACE-4 was observed in 133 (29.0%) patients, including 41 cases (30.4%) in the LDL-C <1.8 mmol/L group, 59 (29.1%) in the LDL-C 1.8–<2.6 mmol/L group, and 33 (27.3%) in the LDL-C ≥2.6 mmol/L group. Compared with the baseline LDL-C <1.8 mmol/L group, the risk of MACE-4 was not statistically different in the LDL-C 1.8–<2.6 mmol/L (HR = 0.90, 95% CI, 0.61–1.35; *P* = 0.620) and ≥2.6 mmol/L groups (HR = 0.86, 95% CI, 0.54–1.36; *P* = 0.521) (Figure 1). After adjusted for multiple covariates including age, gender, comorbidities, baseline Lp(a), secondary prevention medication, on-pump surgery, and the use of internal mammary artery (IMA), compared with patients with baseline LDL-C <1.8 mmol/L, the HR for MACE-4 occurrence was 0.82 (95% CI, 0.51–1.33; *P* = 0.613) for patients with LDL-C 1.8–<2.6 and 0.90 (95% CI, 0.60–1.35; *P* = 0.426) for patients with LDL-C ≥2.6 mmol/L.

**Table 1:** Baseline characteristics of the study population stratified by LDL-C levels.

Characteristics	Total (*N* = 459)	Baseline LDL-C (mmol/L)			*P**-***value	Postoperative LDL-C (mmol/L)			*P**-***value
		LDL-C < 1.8 (*n* = 135)	1.8 **≤** LDL-C < 2.6 (*n* = 203)	LDL-C ≥ 2.6 (*n* = 121)		LDL-C < 1.8 (*n* = 129)	1.8 **≤** LDL-C < 2.6 (*n* = 186)	LDL-C ≥ 2.6 (*n* = 144)	
Age, years, mean (SD)	63.2 (8.1)	63.9 (8.3)	63.9 (8.1)	61.2 (7.9)	0.007	63.3 (8.1)	64.5 (7.9)	61.4 (8.2)	0.002
Male, *n* (%)	374 (81.5)	118 (87.4)	162 (79.8)	94 (77.7)	0.097	113 (87.6)	155 (83.3)	106 (73.6)	0.009
Clinical status, *n* (%)					0.051				0.091
CCS	159 (34.6)	58 (43.0)	62 (30.5)	39 (32.2)		53 (41.1)	65 (35.0)	41 (28.5)	
ACS	300 (65.4)	77 (57.0)	141 (69.5)	82 (67.8)		76 (58.9)	121 (65.1)	103 (71.5)	
Medical history, *n* (%)									
Myocardial infarction	143 (31.2)	40 (29.6)	75 (36.9)	28 (23.1)	0.031	42 (32.6)	59 (31.7)	42 (29.2)	0.814
Hypertension	348 (75.8)	113 (83.7)	151 (74.4)	84 (69.4)	0.023	99 (76.7)	148 (79.6)	101 (70.1)	0.134
Diabetes mellitus	211 (46.0)	68 (50.4)	95 (46.8)	48 (39.7)	0.218	67 (51.9)	88 (47.3)	56 (38.9)	0.087
Peripheral artery disease	79 (17.2)	30 (22.2)	28 (13.8)	21 (17.4)	0.132	20 (15.5)	30 (16.1)	29 (20.1)	0.527
Stroke	52 (11.3)	20 (14.8)	22 (10.8)	10 (8.3)	0.245	15 (11.6)	24 (12.9)	13 (9.0)	0.541
Chronic kidney disease	8 (1.7)	1 (0.7)	5 (2.5)	2 (1.7)	0.599	2 (1.6)	3 (1.6)	3 (2.1)	1.000
Cigarette smoker, *n* (%)	225 (49.0)	72 (53.3)	92 (45.3)	61 (50.4)	0.331	65 (50.4)	93 (50.0)	67 (46.5)	0.769
NYHA, *n* (%)					0.859				0.266
I + II	278 (60.6)	87 (64.4)	121 (59.6)	70 (57.9)		83 (64.3)	115 (61.8)	80 (55.6)	
III + IV	181 (39.4)	48 (35.6)	82 (40.4)	51 (42.1)		46 (35.7)	71 (38.2)	64 (44.4)	
LVEF, *n* (%)					0.602				0.920
<40%	5 (1.1)	0	3 (1.5)	2 (1.7)		1 (0.8)	2 (1.1)	2 (1.4)	
40%–49%	41 (8.9)	11 (8.1)	20 (9.9)	10 (8.3)		13 (10.1)	15 (8.1)	13 (9.0)	
≥50%	413 (90.2)	125 (92.6)	180 (88.7)	108 (90.0)		115 (89.2)	169 (90.9)	129 (89.6)	
SYNTAX score, *n* (%)					0.296				0.646
Low (0–22)	66 (14.4)	14 (10.4)	33 (16.3)	19 (15.7)		15 (11.7)	29 (15.6)	22 (15.3)	
Medium (23–32)	254 (55.3)	80 (59.3)	103 (50.7)	71 (58.7)		72 (55.8)	98 (52.7)	84 (58.3)	
High (≥33)	139 (30.3)	41 (30.4)	67 (33.0)	31 (25.6)		42 (32.6)	59 (31.7)	38 (26.4)	
EuroSCORE, *n* (%)					0.244				0.904
Low (0–2)	183 (39.9)	53 (39.3)	79 (38.9)	51 (42.2)		52 (40.3)	74 (39.8)	57 (39.6)	
Medium (3–5)	210 (45.8)	64 (47.4)	87 (42.9)	59 (48.8)		59 (45.7)	82 (44.1)	69 (47.9)	
High (≥6)	66 (14.4)	18 (13.3)	37 (18.2)	11 (9.1)		18 (14.0)	30 (16.1)	18 (12.5)	
Lp(a) levels (mg/dL), *n* (%)					0.103				0.051
<30	328 (71.5)	103 (76.3)	147 (72.4)	78 (64.5)		102 (79.1)	132 (71.0)	94 (65.3)	
≥30	131 (28.5)	32 (23.7)	56 (27.6)	43 (35.5)		27 (20.9)	54 (29.0)	50 (34.7)	
Antiplatelet therapy, *n* (%)					0.838				0.781
Aspirin	151 (32.9)	45 (33.3)	68 (33.5)	38 (31.4)		42 (32.6)	56 (30.1)	53 (36.8)	
Aspirin + Ticagrelor	159 (34.6)	50 (37.0)	65 (32.0)	44 (36.4)		46 (35.7)	67 (36.0)	46 (31.9)	
Ticagrelor	149 (32.5)	40 (29.6)	70 (34.5)	39 (32.2)		41 (31.8)	63 (33.9)	45 (31.3)	
Medication at discharge, *n* (%)									
Beta-blocker	416 (90.6)	130 (96.3)	181 (89.2)	105 (86.8)	0.055	123 (95.3)	166 (89.2)	127 (88.2)	0.132
ACEI/ARB	274 (59.7)	96 (71.1)	116 (57.1)	62 (51.2)	0.006	83 (64.3)	112 (60.2)	79 (54.9)	0.328
Statin	437 (95.2)	129 (95.6)	194 (95.6)	114 (94.2)	0.373	125 (96.9)	175 (94.1)	137 (95.1)	0.639
Surgical characteristics, *n* (%)									
On-pump	103 (22.4)	19 (14.1)	45 (22.2)	39 (32.2)	0.002	16 (12.4)	42 (22.6)	45 (31.3)	0.001
IMA user	383 (83.4)	107 (79.3)	170 (83.7)	106 (87.6)	0.198	109 (84.5)	153 (82.3)	121 (84.0)	0.849

ACEI, angiotensin-converting enzyme inhibitor; ACS, acute coronary syndrome; ARB, angiotensin receptor blocker; CCS, Canadian Cardiovascular Society; EuroSCORE, European system for cardiac operative risk evaluation; IMA, internal mammary artery; LVEF, left ventricular ejection fraction; NYHA, New York Heart Association functional classification; SYNTAX, synergy between percutaneous coronary intervention with taxus and cardiac surgery; Lp(a), lipoprotein(a).

The results of MACE-3 and MACE-5 yielded similar trends (Figure 1). Compared with the baseline LDL-C <1.8 mmol/L group, the adjusted HR for MACE-3 was 0.82 (95% CI, 0.51–1.33) and 0.80 (95% CI, 0.46–1.37) for the LDL-C 1.8–<2.6 and ≥2.6 mmol/L groups, respectively, and the adjusted HR for MACE-5 was 0.86 (95% CI, 0.58–1.28) and 0.78 (95% CI, 0.49–1.25; all *P* > 0.05), respectively.

### Postoperative LDL-C and clinical outcomes

**Figure 2:**
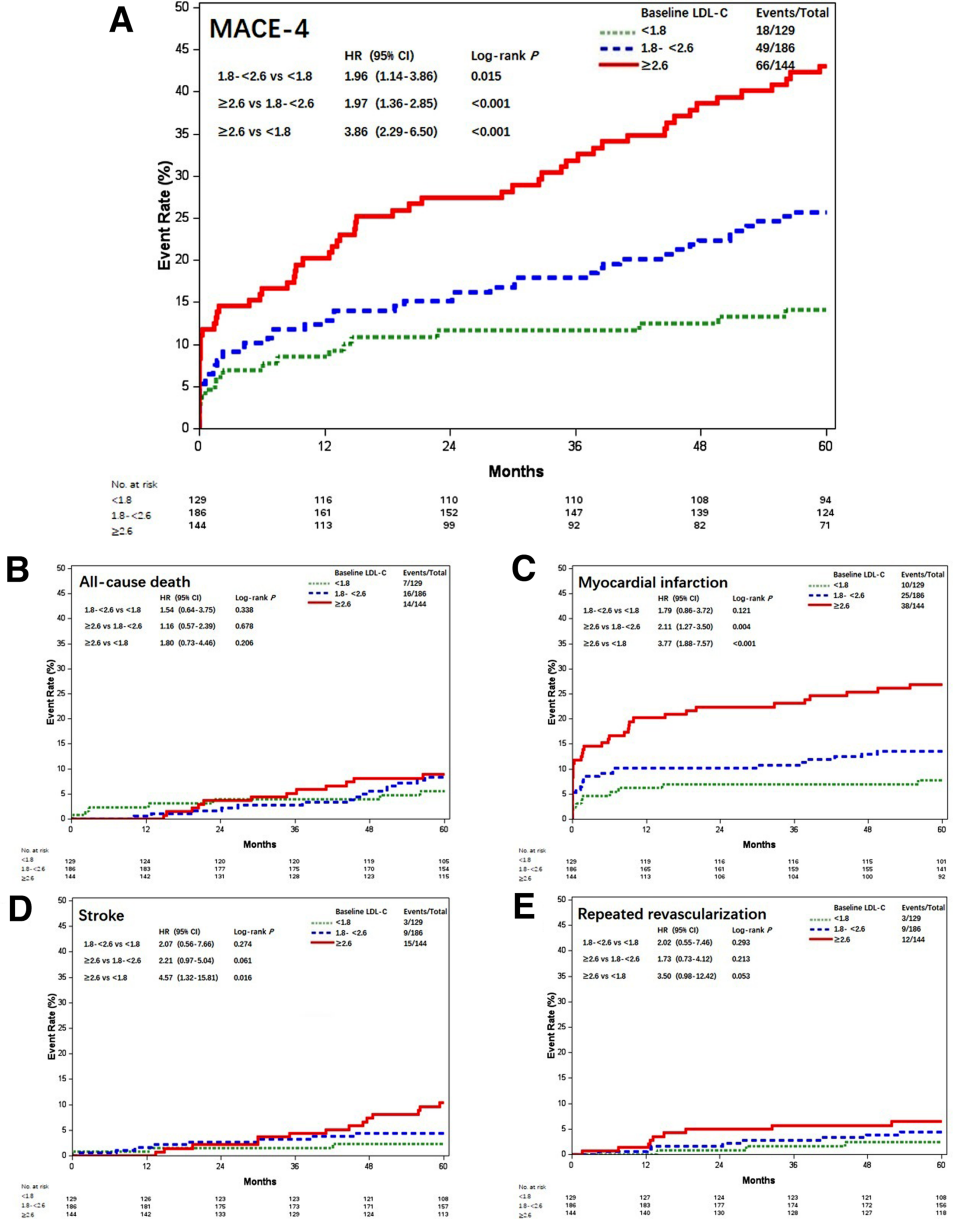
Kaplan–Meier curves of MACE-4 and each individual component among different postoperative LDL-C levels (mmol/L). Kaplan–Meier estimates for freedom from (**A**) MACE-4, (**B**) all-cause death, (**C**) myocardial infarction, (**D**) stroke, and (**E**) repeated revascularization. LDL-C, low-density lipoprotein cholesterol; MACE-4, 4-point major adverse cardiovascular events.

According to the follow-up protocol, repeated measurement of LDL-C levels at 1, 3, 6, and every 12 months after CABG was accomplished. The average LDL-C value of each patient was calculated and the patients were stratified into three groups by referring to the same thresholds. The attainment of LDL-C target <1.8 mmol/L following CABG was accomplished in 129 (28.1%) patients, 186 (40.5%) achieved LDL-C 1.8–<2.6 mmol/L, and 144 (31.4%) remained with LDL-C ≥2.6 mmol/L. Most characteristics were comparable, except that patients with increased postoperative LDL-C were younger, lower male gender, and more associated with on-pump CABG (Table 1). MACE-4 was reported in 18 (14.0%) patients with LDL-C <1.8 mmol/L, 49 (26.3%) with LDL-C 1.8–<2.6 mmol/L, and 66 (45.8%) with LDL-C ≥2.6 mmol/L. Compared with the postoperative LDL-C <1.8 mmol/L group, the risk of MACE-4 was higher for patients with LDL-C 1.8–<2.6 and ≥2.6 mmol/L. The crude HRs were 1.96 (95% CI, 1.14–3.86; *P* = 0.015) and 3.86 (95% CI, 2.29–6.50; *P* < 0.001) (Figure 2). After adjusted for age, gender, comorbidities, baseline LDL-C, Lp(a), secondary prevention medication, on-pump surgery, and use of IMA, the HRs for MACE-4 occurrence were 1.92 (95% CI, 1.12–3.29; *P* = 0.019) and 3.90 (95% CI, 2.29–6.64; *P* < 0.001) for LDL-C 1.8–<2.6 and ≥2.6 mmol/L groups, when compared with the LDL-C <1.8 mmol/L group. Postoperative LDL-C ≥2.6 mmol/L was mainly associated with increased risk of MI and stroke (all *P* < 0.05), but not in all-cause death and repeated .revascularization (all *P* > 0.05) compared with LDL-C <1.8 mmol/L (Figure 2).

**Figure 3:**
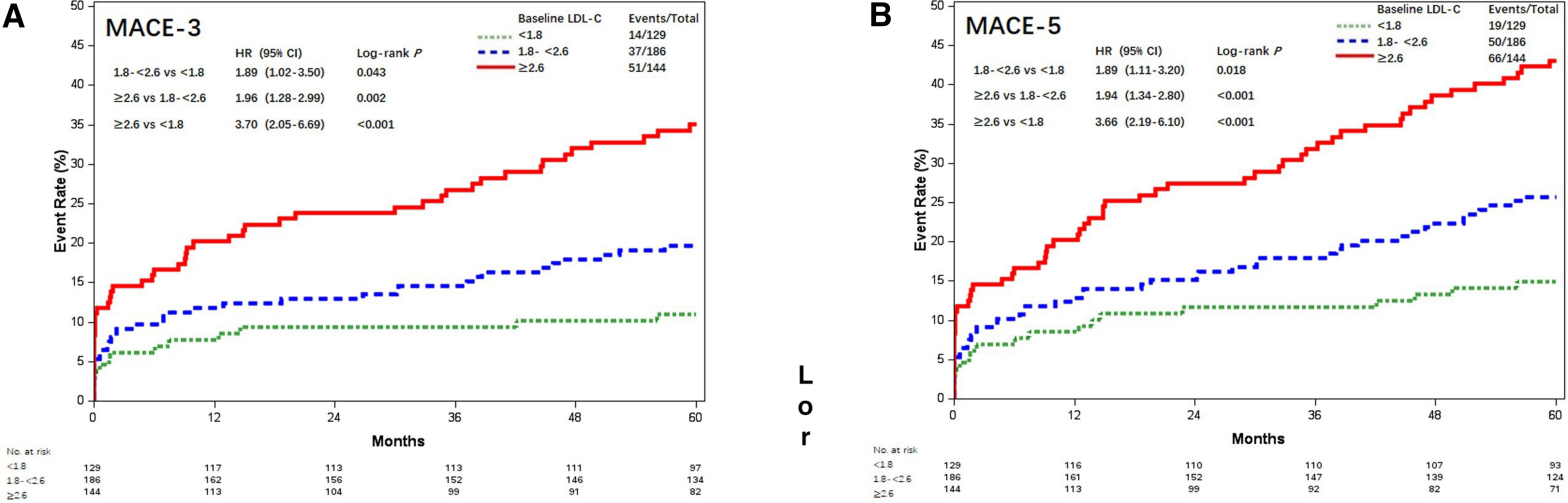
Kaplan–Meier curves of MACE-4 and MACE-5 among different postoperative LDL-C levels (mmol/L). Kaplan–Meier estimates for freedom from (**A**) MACE-3 and (**B**) MACE-5. LDL-C, low-density lipoprotein cholesterol; MACE-4, 4-point major adverse cardiovascular events; MACE-3, 3-point major adverse cardiovascular events; MACE-5, 5-point major adverse cardiovascular events.

The results of MACE-3 and MACE-5 were similar (Figure 3). Compared with patients with postoperative LDL-C <1.8 mmol/L, the adjusted HR for MACE-3 was 1.86 (95% CI, 1.00–3.45; *P* = 0.048) and 3.61 (95% CI, 1.98–6.58; *P* < 0.001) for patients with LDL-C 1.8–<2.6 and LDL-C ≥2.6 mmol/L; the adjusted HR for MACE-5 was 1.85 (95% CI, 1.09–3.14; *P* = 0.023) and 3.65 (95% CI, 2.17–6.14; *P* < 0.001), respectively.

### Lp(a) and clinical outcomes

**Figure 4:**
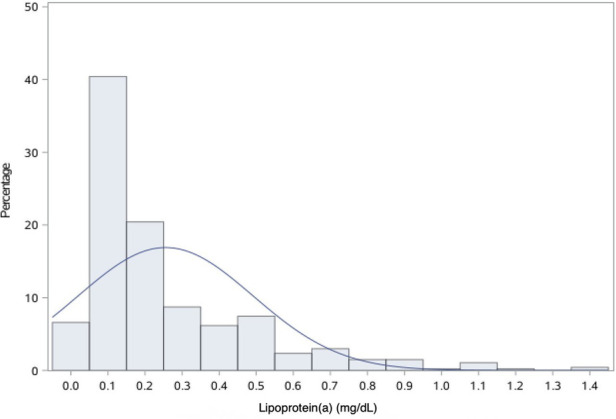
Distribution of Lp(a). Lp(a), lipoprotein(a).

**Figure 5:**
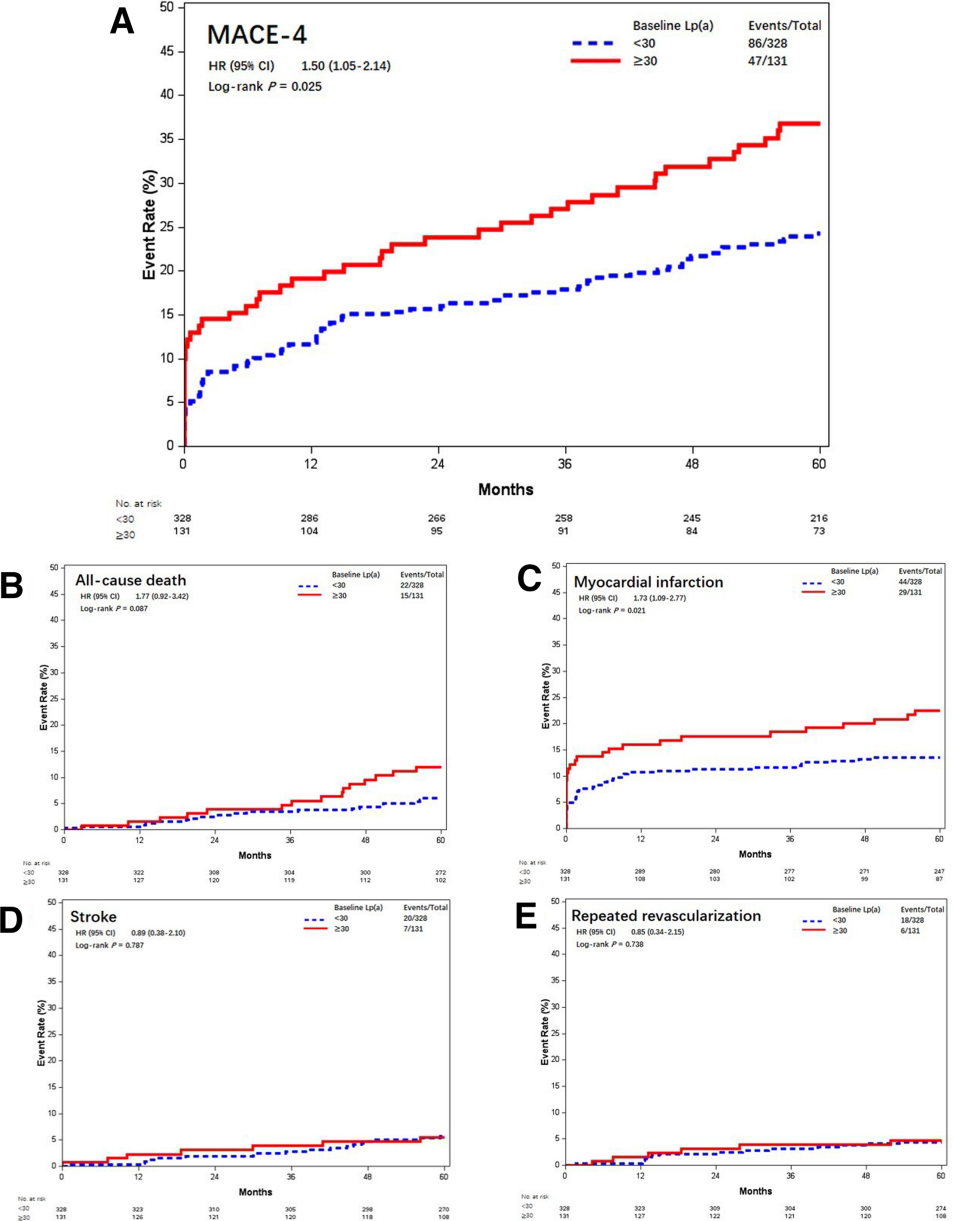
Kaplan–Meier curves of MACE-4 and each individual component different baseline Lp(a) levels (mmol/L). Kaplan–Meier estimates for freedom from (**A**) MACE-4, (**B**) all-cause death, (**C**) myocardial infarction, (**D**) stroke, and (**E**) repeated revascularization. Lp(a), lipoprotein(a); MACE-4, 4-point major adverse cardiovascular events.

In this study cohort, Lp(a) ranged from 1 to 138 mg/dL, with a median level of 17 mg/dL (interquartile range, 9–31 mg/dL). The distribution of Lp(a) was skewed and Lp(a) ≥30 mg/dL was identified in 131 (28.5%) patients (Figure 4). Most baseline characteristics between the two groups were comparable, except that patients with Lp(a) ≥30 mg/dL had a lower prevalence of hypertension (Table 2). MACE-4 was detected in 47 patients in the Lp(a) ≥30 mg/dL group and in 86 in the Lp(a) <30 mg/dL group. As shown by the Kaplan–Meier curves, compared with the Lp(a) <30 mg/dL group, patients with Lp(a) ≥30 mg/dL had a higher incidence of MACE-4 (35.9% vs. 26.2%; *P* = 0.024) with a crude HR of 1.50 (95% CI, 1.05–2.14; *P* = 0.025) (Figure 5). The adjusted HR (aHR) (adjusted for age, gender, comorbidities, baseline LDL-C, secondary prevention medication, on-pump surgery, and use of IMA) was 1.52 (95% CI, 1.06–2.18; *P* = 0.022) for MACE-4. The increased risk of MACE-4 was mainly due to the increased risk of MI in the Lp(a) ≥30 mg/dL group (22.1% vs. 13.4%, HR = 1.73, 95% CI, 1.09–2.77; *P* = 0.021). No significant difference was found concerning the risk of all-cause death (11.5% vs. 6.7%, HR = 1.77, 95% CI, 0.92–3.42; *P* = 0.087), stroke (5.3% vs. 6.1%, HR = 0.89, 95% CI, 0.38–2.10; *P* = 0.787), and repeated revascularization (4.6% vs. 5.5%, HR = 0.85, 95% CI, 0.34–2.15; *P* = 0.738) between the two groups (Figure 5).

**Table 2:** Baseline characteristics of the study population stratified by baseline Lp(a) levels.

Characteristics	Lp(a) <30 mg/dL (*n* = 328)	Lp(a) ≥30 mg/dL (*n* = 131)	*P-*value
Age, years, mean (SD)	63.4 (8.3)	62.8 (7.8)	0.487
Male, *n* (%)	267 (81.4)	107 (81.7)	0.945
Clinical status, *n* (%)			0.605
CCS	111 (33.8)	48 (36.6)	
ACS	217 (66.2)	83 (63.4)	
Medical history, *n* (%)			
Myocardial infarction	96 (29.3)	47 (35.9)	0.167
Hypertension	260 (79.3)	88 (67.2)	0.006
Diabetes mellitus	158 (48.2)	53 (40.5)	0.134
Peripheral artery disease	54 (16.5)	25 (19.1)	0.502
Stroke	39 (11.9)	13 (9.9)	0.548
Chronic kidney disease	6 (1.8)	2 (1.5)	1.000
Cigarette smoker, *n* (%)	158 (48.2)	67 (51.2)	0.565
NYHA, *n* (%)			0.599
I + II	203 (61.9)	75 (57.3)	
III + IV	125 (38.1)	56 (42.7)	
LVEF, *n* (%)			0.636
<40%	3 (0.9)	2 (1.5)	
40%–49%	27 (8.2)	14 (10.7)	
≥50%	298 (90.9)	115 (87.8)	
SYNTAX score, *n* (%)			0.820
Low (0–22)	46 (14.0)	20 (15.3)	
Medium (23–32)	180 (54.9)	74 (56.5)	
High (≥33)	102 (31.1)	37 (28.2)	
EuroSCORE, *n* (%)			0.470
Low (0–2)	133 (40.6)	50 (38.2)	
Medium (3–5)	152 (46.3)	58 (44.3)	
High (≥6)	43 (13.1)	23 (17.6)	
Baseline LDL-C levels (mmol/L), *n* (%)			0.103
<1.8	103 (31.4)	32 (24.3)	
1.8–<2.6	147 (44.8)	56 (43.8)	
≥2.6	78 (23.8)	43 (32.8)	
Antiplatelet therapy, *n* (%)			0.547
Aspirin	112 (34.2)	39 (29.8)	
Aspirin + ticagrelor	114 (34.8)	45 (34.4)	
Ticagrelor	102 (31.1)	47 (35.9)	
Medication at discharge, *n* (%)			
Beta-blocker	295 (89.9)	121 (92.4)	0.382
ACEI/ARB	201 (61.3)	73 (55.7)	0.268
Statin	310 (94.5)	127 (96.9)	0.251
Surgical characteristics, *n* (%)			
On-pump	73 (22.3)	30 (22.9)	0.699
IMA user	278 (84.8)	105 (80.2)	0.231

ACEI, angiotensin-converting enzyme inhibitor; ACS, acute coronary syndrome; ARB, angiotensin receptor blocker; CCS, Canadian Cardiovascular Society; EuroSCORE, European system for cardiac operative risk evaluation; IMA, internal mammary artery; LVEF, left ventricular ejection fraction; NYHA, New York Heart Association functional classification; SYNTAX, synergy between percutaneous coronary intervention with taxus and cardiac surgery; Lp(a), lipoprotein(a); LDL-C, low-density lipoprotein cholesterol.

**Figure 6:**
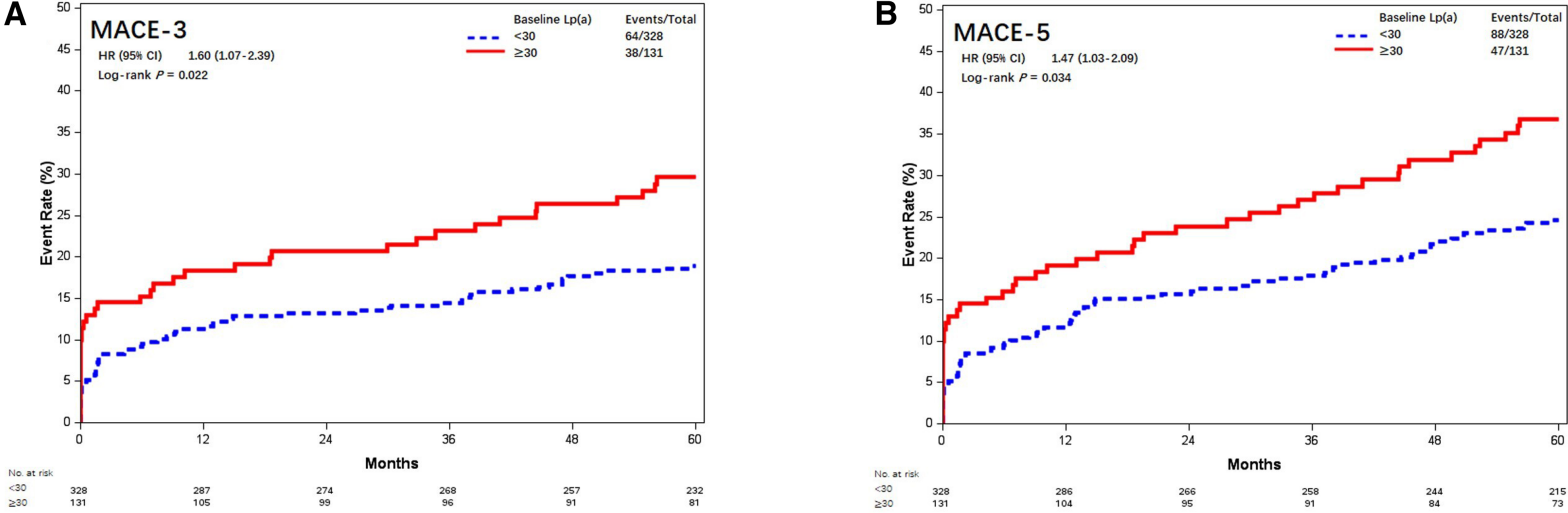
Kaplan–Meier curves of MACE-3 and MACE-5 between different baseline Lp(a) levels (mmol/L). Kaplan–Meier estimates for freedom from (**A**) MACE-3 and (**B**) MACE-5. Lp(a), lipoprotein(a); MACE-3, 3-point major adverse cardiovascular events; MACE-5, 5-point major adverse cardiovascular events.

Similarly, Lp(a) ≥30 mg/dL was associated with increased risk of MACE-3 (29.0% vs. 19.5%, aHR = 1.62, 95% CI, 1.08–2.44; *P* = 0.019) and MACE-5 (35.9% vs. 26.8%, aHR = 1.49, 95% CI, 1.04–2.14; *P* = 0.028) (Figure 6).

### 1-year statin adherence and clinical outcomes

Information about statin use was updated every 3 months during the first year after CABG. A total of 437 (95.2%) patients were reported to be dispensed with statins constantly and 22 patients discontinued the use of statin. Baseline characteristics between the two groups were compared. Poor 1-year statin adherence was observed more in patients with increased postoperative LDL-C levels, receiving dual antiplatelet therapy, and less use of IMA (Supplementary Table S1). MACE-4 occurred in 10 (45.5%) patients with poor adherence and 123 (28.1%) patients with good adherence. The crude HR for MACE-4 was 2.03 (95% CI, 1.07–3.88; *P* = 0.031) (Supplementary Figure S1). Poor 1-year statin adherence was associated with increased MACE-4 events with an adjusted HR of 2.32 (95% CI, 1.19–4.52; *P* = 0.013). Increased risk of MACE-4 was mainly due to the increased risk of all-cause death (22.7% vs. 7.3%, HR = 3.92, 95% CI, 1.52–10.09; *P* = 0.005) (Supplementary Figure S2). Poor 1-year statin adherence was also related with enhanced risk of MACE-5 (50.0% vs. 28.4%, aHR = 2.62, 95% CI, 1.38–4.98; *P* = 0.003), but not with MACE-3 (31.8% vs. 21.7%, aHR = 2.04, 95% CI, 0.93–4.51; *P* = 0.077) (Supplementary Figure S1).

## Discussion

Our main findings delineated that postoperative LDL-C was significantly associated with 5-year MACE, but no relationship was observed between baseline concentration and MACE. In addition, baseline Lp(a) ≥30 mg/dl was also independently related with increased risk of 5-year MACE. Reinforcing routine measurement of Lp(a) and postoperative LDL-C, promoting the attainment of LDL-C target, and prescribing combined lipid-lowering therapy aiming at multiple pathways should be encouraged in CABG patients.

CABG surgery is widely used to effectively improve prognosis in patients with complex and severe CAD; however, patients following CABG are still susceptible to high risk of subsequent adverse events when compared with the general population (21). It was reported that within 5 years after CABG, 20%–30% patients experienced MACE and 15% died (22, 23). Due to the high residual risk of cardiovascular morbidity and mortality, implantation and optimization of postoperative secondary prevention is highly recommended (24, 25). Management of lipid abnormalities remains one of the primary goals in secondary prevention, for the reason that hyperlipidemia is associated with increased future cardiovascular events and graft deterioration (26, 27). Despite the benefit of lipid-lowering therapy, its underuse is common and compliance with lipid-lowering medication continues to decline over time (10, 28). Compared with patients receiving PCI, fulfillment of statin prescription and maintenance of statin use were markedly unsatisfactory in the CABG population (11, 29). Meanwhile, the overall attainment of LDL-C target in the secondary prevention setting was low, which implied a huge gap between real-world data and guideline recommendation (30).

In this CABG cohort, 40.5% of the patients with LDL-C 1.8–<2.6 mmol/L and 31.4% with LDL-C still remaining ≥2.6 mmol/L were proved to be encountered with increased risk of MACE. Meanwhile, only 28.1% of patients achieved the goal of LDL-C <1.8 mmol/L during the 5-year follow-up period. Likewise, under-implementation of the guideline recommended LDL-C target in the CABG population is common. Results from 1-year follow-up of a Middle-Eastern cohort showed that 59.3% and 29% of CABG patients attained LDL-C targets of <1.8 mmol/L and 1.4 mmol/L, respectively (31). A most recent study from Australia with a median follow-up of 483 days reported that the attainment of LDL-C target <1.8 mmol/L was 47.7%and that of <1.4 mmol/L was 24.4% (32). Another retrospective analysis of 1,230 CABG patients with a median follow-up period of 101 months showed that 44% of the patients reached the target of <1.8 mmol/L (33). A single-center study with a median follow-up time of 12.5 years from Hong Kong had similar results that less than 25% of patients with statins were able to achieve the LDL-C target (34). Thus, there was much room for improvement of postoperative LDL-C control in CABG patients.

Lipid target including LDL-C was not only a treatment goal for lipid-lowering therapy but also a predictive factor for risk stratification in patients following CABG. A large randomized controlled study recruited 10,001 patients composed of 4,654 patients with a history of CABG and found that aggressive LDL reduction to a mean of 2.0 mmol/L was associated with reduced MACE (nonfatal MI, cardiac death, resuscitated cardiac arrest, or stroke) of 27% compared with patients who underwent standard LDL-C reduction to a mean of 2.6 mmol/L, and lowered the need for revascularization rate by 30% (35). Zafrir et al. found that uncontrolled postoperative LDL-C was independently associated with decreased survival, compared with LDL-C <1.8 mmol/L, and the HR for long-term mortality was 1.33 and 1.97 for patients with LDL-C 1.8–2.6 mmol/L and >2.6 mmol/L, respectively (33). A recent meta-analysis proved that intensive LDL-C reduction was associated with 14% relative reduction in all-cause deaths and 25% relative reduction in CV deaths in CABG patients, which highlighted mortality benefits (36). Altogether, exposure to increased postoperative LDL-C was correlated with higher cardiovascular risk.

Results from several observational studies proved that statin use was associated with improved survival and reduced MACE occurrence after CABG (37, 38). In the present analysis, we focused on exploring the influence of statin adherence and found that poor adherence during the first year was correlated with increased risk of MACE, which suggested that CABG patients could benefit from long-term continuous statin use. With the development of non-statin lipid-lowering therapy, we had entered into a new era of hyperlipidemia management. It had been proved that patients with CABG history derive larger absolute reductions in MACE and death from PCSK9 inhibitor (9). Whether early initiation of non-statin LDL-C-targeted lipid-lowering therapy could further reduce subsequent cardiovascular risk and vein graft disease in patients with recent CABG procedure warrants further investigation.

Although LDL-C remained the prime target and statins continued to be the mainstay of secondary prevention, the attribution of Lp(a) to cardiovascular risk was also established. Results from a meta-analysis proved that patients with confirmed CVD and raised Lp(a) concentration were at substantial residual risk, even while taking statins, and Lp(a)-targeted lowering therapy might succeed in reducing Lp(a)-mediated risk (17). Lp(a) was underestimated and infrequently measured in the CABG population. Results from a prospective single-center research with a mean follow-up duration of 8.5 years showed that Lp(a) ≥30 mg/dL was identified in 39% of the CABG subjects and Lp(a) ≥30 mg/dL was significantly associated with greater risk of the composite outcome of CV death and nonfatal MI with HR = 2.98 compared with patients with Lp(a) <30 mg/dL (39). In the DACAB trial, 92% of the CABG population had Lp(a) tested and 28.5% of them had Lp(a) ≥30 mg/dL. We observed that patients with Lp(a) ≥30 mg/dL had a greater risk for the 5-year MACE occurrence. Longer follow-up duration and marked Lp(a) variations between ethnic groups might lead to disparity of these results. In addition, it was reported that Lp(a) accumulation in graft vessel contributed to early occlusion of vein grafts and the adoption of lipoprotein apheresis significantly improved vein graft patency during the first year after CABG (14, 40). In addition, a large meta-analysis indicated that most statins may increase Lp(a) by an average of 8%–24% or 11% increase in the geometric mean of Lp(a) (41). The current research highlighted that in order to better predict residual risk in CABG patients, use of LDL-C as a single biomarker might not be appropriate. Elevated Lp(a) levels contributed to residual risk in the CABG population; thus, new targeted therapy could potentially mitigate future cardiovascular events.

### Study limitations

There were several limitations in our research that should be taken into account. First, due to prospective observational study essence, bias could not be avoided and additional confounding factors might not be detected and adjusted, but our research was based on a well-conducted randomized trial, and LDL-C and Lp(a) levels were prospectively collected. Second, we did not evaluate the long-term adherence of statins and did not investigate the utilization of ezetimibe or PCSK9 inhibitor during the follow-up, but statin monotherapy predominated as the mode of lipid management in this cohort and the first PCSK9 inhibitor was not approved by the Chinese CFDA until 2018. Third, Lp(a) was reported in mass concentration instead of particle concentration, which might lead to over- or underestimation of the true Lp(a) burden due to the size heterogeneity of the apo(a) component. Fourth, other lipid profiles including triglyceride levels were not collected and investigated in this current analysis. Fifth, this secondary analysis was based on the Chinese population; a cautious interpretation is warranted when the results were generalized to other ethnic groups.

## Conclusion

Exposure to uncontrolled postoperative LDL-C or high Lp(a) was associated with an increased risk of 5-year MACE after CABG. More stringent management of LDL-C and promotion of statin adherence are essential. Combined lipid-lowering therapy incorporating novel agents targeted at new causal lipoproteins merits further research.

## Data Availability

All data that underlie the results reported in this article will be provided upon reasonable request. Requests to access these datasets should be directed to the corresponding author.

## References

[1] Gaudino M Taggart D Suma H Puskas JD Crea F Massetti M. The choice of conduits in coronary artery bypass surgery. J Am Coll Cardiol. (2015) 66(15):1729–37. 10.1016/j.jacc.2015.08.395 26449144

[2] Lawton JS Tamis-Holland JE Bangalore S Bates ER Beckie TM Bischoff JM. 2021 ACC/AHA/SCAI guideline for coronary artery revascularization: a report of the American College of Cardiology/American Heart Association Joint Committee on Clinical Practice Guidelines. J Am Coll Cardiol. (2022) 79(2):e21–129 [published correction appears in *J Am Coll Cardiol*. (2022) **79**(15):1547]. 10.1016/j.jacc.2021.09.006 34895950

[3] Neumann FJ Sousa-Uva M Ahlsson A Alfonso F Banning AP Benedetto U. 2018 ESC/EACTS guidelines on myocardial revascularization. Eur Heart J. (2019) 40(2):87–165 [published correction appears in *Eur Heart J*. (2019) **40**(37):3096]. 10.1093/eurheartj/ehy394 30165437

[4] Yahagi K Kolodgie FD Otsuka F Finn AV Davis HR Joner M. Pathophysiology of native coronary, vein graft, and in-stent atherosclerosis. Nat Rev Cardiol. (2016) 13(2):79–98. 10.1038/nrcardio.2015.164 26503410

[5] Kulik A Ruel M Jneid H Ferguson TB Hiratzka LF Ikonomidis JS. Secondary prevention after coronary artery bypass graft surgery: a scientific statement from the American Heart Association. Circulation. (2015) 131(10):927–64. 10.1161/CIR.0000000000000182 25679302

[6] Naci H Brugts JJ Fleurence R Tsoi B Toor H Ades AE. Comparative benefits of statins in the primary and secondary prevention of major coronary events and all-cause mortality: a network meta-analysis of placebo-controlled and active-comparator trials. Eur J Prev Cardiol. (2013) 20(4):641–57. 10.1177/2047487313480435 23447425

[7] Kurlansky P Herbert M Prince S Mack M. Coronary artery bypass graft versus percutaneous coronary intervention: meds matter: impact of adherence to medical therapy on comparative outcomes. Circulation. (2016) 134(17):1238–46. 10.1161/CIRCULATIONAHA.115.021183 27777293

[8] Pinho-Gomes AC Azevedo L Ahn JM Park SJ Hamza TH Farkouh ME. Compliance with guideline-directed medical therapy in contemporary coronary revascularization trials. J Am Coll Cardiol. (2018) 71(6):591–602. 10.1016/j.jacc.2017.11.068 29420954

[9] Goodman SG Aylward PE Szarek M Chumburidze V Bhatt DL Bittner VA. Effects of alirocumab on cardiovascular events after coronary bypass surgery. J Am Coll Cardiol. (2019) 74(9):1177–86. 10.1016/j.jacc.2019.07.015 31466614

[10] Kulik A Levin R Ruel M Mesana TG Solomon DH Choudhry NK. Patterns and predictors of statin use after coronary artery bypass graft surgery. J Thorac Cardiovasc Surg. (2007) 134(4):932–8. 10.1016/j.jtcvs.2007.05.039 17903510

[11] Hlatky MA Solomon MD Shilane D Leong TK Brindis R Go AS. Use of medications for secondary prevention after coronary bypass surgery compared with percutaneous coronary intervention. J Am Coll Cardiol. (2013) 61(3):295–301. 10.1016/j.jacc.2012.10.018 23246391

[12] Kronenberg F Mora S Stroes ESG Ference BA Arsenault BJ Berglund L. Lipoprotein(a) in atherosclerotic cardiovascular disease and aortic stenosis: a European Atherosclerosis Society consensus statement. Eur Heart J. (2022) 43(39):3925–46. 10.1093/eurheartj/ehac361 36036785 PMC9639807

[13] Duarte Lau F Giugliano RP. Lipoprotein(a) and its significance in cardiovascular disease: a review. JAMA Cardiol. (2022) 7(7):760–9 [published correction appears in *JAMA Cardiol*. (2022) **7**(7):776]. 10.1001/jamacardio.2022.0987 35583875

[14] Nordestgaard BG Chapman MJ Ray K Borén J Andreotti F Watts GF. Lipoprotein(a) as a cardiovascular risk factor: current status. Eur Heart J. (2010) 31(23):2844–53. 10.1093/eurheartj/ehq386 20965889 PMC3295201

[15] Madsen CM Kamstrup PR Langsted A Varbo A Nordestgaard BG. Lipoprotein(a)-lowering by 50 mg/dl (105 nmol/L) may be needed to reduce cardiovascular disease 20% in secondary prevention: a population-based study. Arterioscler Thromb Vasc Biol. (2020) 40(1):255–66. 10.1161/ATVBAHA.119.312951 31578080

[16] Albers JJ Slee A O'Brien KD Robinson JG Kashyap ML Kwiterovich PO Jr. Relationship of apolipoproteins A-1 and B, and lipoprotein(a) to cardiovascular outcomes: the AIM-HIGH trial (atherothrombosis intervention in metabolic syndrome with low HDL/high triglyceride and impact on global health outcomes). J Am Coll Cardiol. (2013) 62(17):1575–9. 10.1016/j.jacc.2013.06.051 23973688 PMC3800510

[17] Willeit P Ridker PM Nestel PJ Simes J Tonkin AM Pedersen TR. Baseline and on-statin treatment lipoprotein(a) levels for prediction of cardiovascular events: individual patient-data meta-analysis of statin outcome trials. Lancet. (2018) 392(10155):1311–20. 10.1016/S0140-6736(18)31652-0 30293769

[18] Zhao Q Zhu Y Xu Z Cheng Z Mei J Chen X. Effect of ticagrelor plus aspirin, ticagrelor alone, or aspirin alone on saphenous vein graft patency 1 year after coronary artery bypass grafting: a randomized clinical trial. JAMA. (2018) 319(16):1677–86. 10.1001/jama.2018.3197 29710164 PMC5933396

[19] Catapano AL Graham I De Backer G Wiklund O Chapman MJ Drexel H. 2016 ESC/EAS guidelines for the management of dyslipidaemias. Eur Heart J. (2016) 37(39):2999–3058. 10.1093/eurheartj/ehw272 27567407

[20] Tsimikas S. A test in context: lipoprotein(a): diagnosis, prognosis, controversies, and emerging therapies. J Am Coll Cardiol. (2017) 69(6):692–711. 10.1016/j.jacc.2016.11.042 28183512

[21] Adelborg K Horváth-Puhó E Schmidt M Munch T Pedersen L Nielsen PH. Thirty-year mortality after coronary artery bypass graft surgery: a Danish nationwide population-based cohort study. Circ Cardiovasc Qual Outcomes. (2017) 10(5):e002708. 10.1161/CIRCOUTCOMES.116.002708 28500223

[22] Mohr FW Morice MC Kappetein AP Feldman TE Ståhle E Colombo A. Coronary artery bypass graft surgery versus percutaneous coronary intervention in patients with three-vessel disease and left main coronary disease: 5-year follow-up of the randomised, clinical SYNTAX trial. Lancet. (2013) 381(9867):629–38. 10.1016/S0140-6736(13)60141-5 23439102

[23] Stone GW Kappetein AP Sabik JF Pocock SJ Morice MC Puskas J. Five-year outcomes after PCI or CABG for left main coronary disease. N Engl J Med. (2019) 381(19):1820–30 [published correction appears in *N Engl J Med*. (2020) **382**(11):1078]. 10.1056/NEJMoa1909406 31562798

[24] Leviner DB Zafrir B Jaffe R Saliba W Flugelman MY Sharoni E. Impact of modifiable risk factors on long-term outcomes after coronary artery bypass surgery. Thorac Cardiovasc Surg. (2021) 69(7):592–8. 10.1055/s-0040-1719154 33260234

[25] Gaudino M Antoniades C Benedetto U Deb S Di Franco A Di Giammarco G. Mechanisms, consequences, and prevention of coronary graft failure. Circulation. (2017) 136(18):1749–64. 10.1161/CIRCULATIONAHA.117.027597 29084780

[26] Björklund E Nielsen SJ Hansson EC Karlsson M Wallinder A Martinsson A. Secondary prevention medications after coronary artery bypass grafting and long-term survival: a population-based longitudinal study from the SWEDEHEART registry. Eur Heart J. (2020) 41(17):1653–61. 10.1093/eurheartj/ehz714 31638654 PMC7194184

[27] Hiratzka LF Eagle KA Liang L Fonarow GC LaBresh KA Peterson ED. Atherosclerosis secondary prevention performance measures after coronary bypass graft surgery compared with percutaneous catheter intervention and nonintervention patients in the get with the guidelines database. Circulation. (2007) 116(11 Suppl):I207–12. 10.1161/CIRCULATIONAHA.106.681247 17846305

[28] Kotseva K De Backer G De Bacquer D Rydén L Hoes A Grobbee D. Lifestyle and impact on cardiovascular risk factor control in coronary patients across 27 countries: results from the European society of cardiology ESC-EORP EUROASPIRE V registry. Eur J Prev Cardiol. (2019) 26(8):824–35. 10.1177/2047487318825350 30739508

[29] Gitt AK Lautsch D Ferrieres J Kastelein J Drexel H Horack M. Low-density lipoprotein cholesterol in a global cohort of 57,885 statin-treated patients. Atherosclerosis. (2016) 255:200–9. 10.1016/j.atherosclerosis.2016.09.004 27667299

[30] Ray KK Molemans B Schoonen WM Giovas P Bray S Kiru G. EU-wide cross-sectional observational study of lipid-modifying therapy use in secondary and primary care: the DA VINCI study. Eur J Prev Cardiol. (2021) 28(11):1279–89. 10.1093/eurjpc/zwaa047 33580789

[31] Atallah B Khaddage R Sadik ZG Mallah SI Lee-St John TJ Alfardan S. Lipid control post coronary artery bypass graft: one year follow-up of a middle-eastern cohort. Glob Heart. (2020) 15(1):12. 10.5334/gh.530 32489785 PMC7218787

[32] Lan NSR Ali US Yeap BB Fegan PG Larbalestier R Bell DA. Attainment of lipid targets following coronary artery bypass graft surgery: can we do better? J Lipid Atheroscler. (2022) 11(2):187–96. 10.12997/jla.2022.11.2.187 35656149 PMC9133779

[33] Zafrir B Saliba W Jaffe R Sliman H Flugelman MY Sharoni E. Attainment of lipid goals and long-term mortality after coronary-artery bypass surgery. Eur J Prev Cardiol. (2019) 26(4):401–8. 10.1177/2047487318812962 30426772

[34] Lim K Wong CHM Lee ALY Fujikawa T Wong RHL. Influence of cholesterol level on long-term survival and cardiac events after surgical coronary revascularization. JTCVS Open. (2022) 10:195–203. 10.1016/j.xjon.2022.02.022 36004261 PMC9390627

[35] Shah SJ Waters DD Barter P Kastelein JJ Shepherd J Wenger NK. Intensive lipid-lowering with atorvastatin for secondary prevention in patients after coronary artery bypass surgery. J Am Coll Cardiol. (2008) 51(20):1938–43. 10.1016/j.jacc.2007.12.054 18482661

[36] Alkhalil M. Effects of intensive lipid-lowering therapy on mortality after coronary bypass surgery: a meta-analysis of 7 randomised trials. Atherosclerosis. (2020) 293:75–8. 10.1016/j.atherosclerosis.2019.12.006 31865057

[37] Aihara K Miyauchi K Kasai T Kubota N Kajimoto K Tamura H. Long-term efficacy of pravastatin therapy in diabetic patients undergoing complete coronary revascularization. J Atheroscler Thromb. (2010) 17(4):350–5. 10.5551/jat.1925 20154448

[38] Pan E Nielsen SJ Mennander A Björklund E Martinsson A Lindgren M. Statins for secondary prevention and major adverse events after coronary artery bypass grafting. J Thorac Cardiovasc Surg. (2022) 164(6):1875–86.e4. 10.1016/j.jtcvs.2021.08.088 34893327

[39] Ezhov MV Safarova MS Afanasieva OI Kukharchuk VV Pokrovsky SN. Lipoprotein(a) level and apolipoprotein(a) phenotype as predictors of long-term cardiovascular outcomes after coronary artery bypass grafting. Atherosclerosis. (2014) 235(2):477–82. 10.1016/j.atherosclerosis.2014.05.944 24952151

[40] Ezhov MV Afanasieva OI Il'ina LN Safarova MS Adamova IY Matchin YG. Association of lipoprotein(a) level with short- and long-term outcomes after CABG: the role of lipoprotein apheresis. Atheroscler Suppl. (2017) 30:187–92. 10.1016/j.atherosclerosissup.2017.05.011 29096836

[41] Tsimikas S Gordts PLSM Nora C Yeang C Witztum JL. Statin therapy increases lipoprotein(a) levels. Eur Heart J. (2020) 41(24):2275–84. 10.1093/eurheartj/ehz310 31111151

