# Supplementary material for: Impact of low-density lipoprotein cholesterol and lipoprotein(a) on mid-term clinical outcomes following coronary artery bypass grafting: A secondary analysis of the DACAB trial
Source: Front Cardiovasc Med. 2023 Mar 24;10:1103681. doi: 10.3389/fcvm.2023.1103681 (PMC10080087; doi:10.3389/fcvm.2023.1103681)
Supplement: Supplementary file 1 [file Datasheet1.pdf]

Figure E1 Kaplan-Meier curves of MACE according to statin adherence

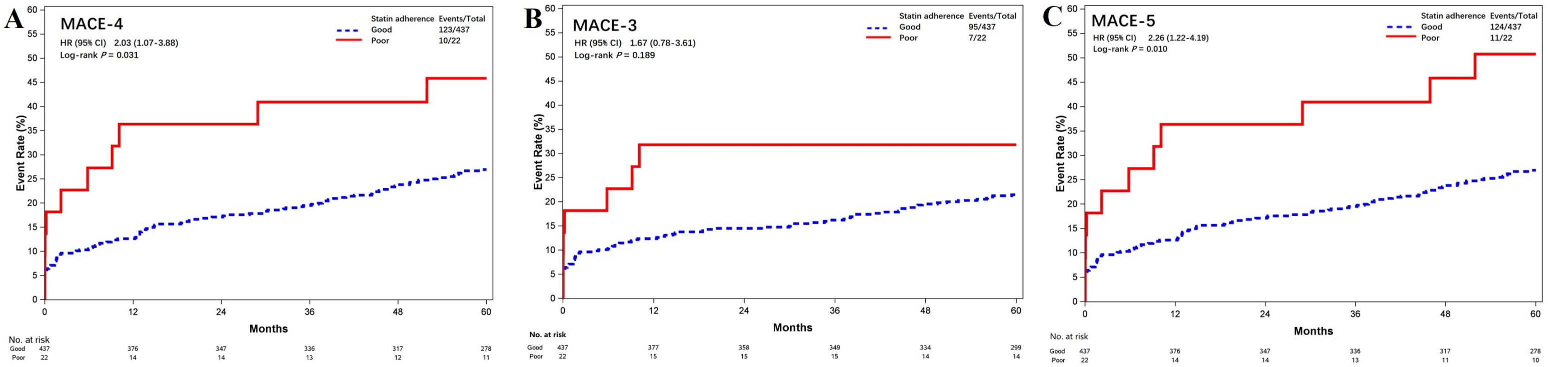

Kaplan-Meier estimates for freedom from **(A)** MACE-4, **(B)** MACE-3, **and (C)** MACE-5.

Figure E2 Kaplan-Meier curves of each individual component of MACE-4 according to statin adherence

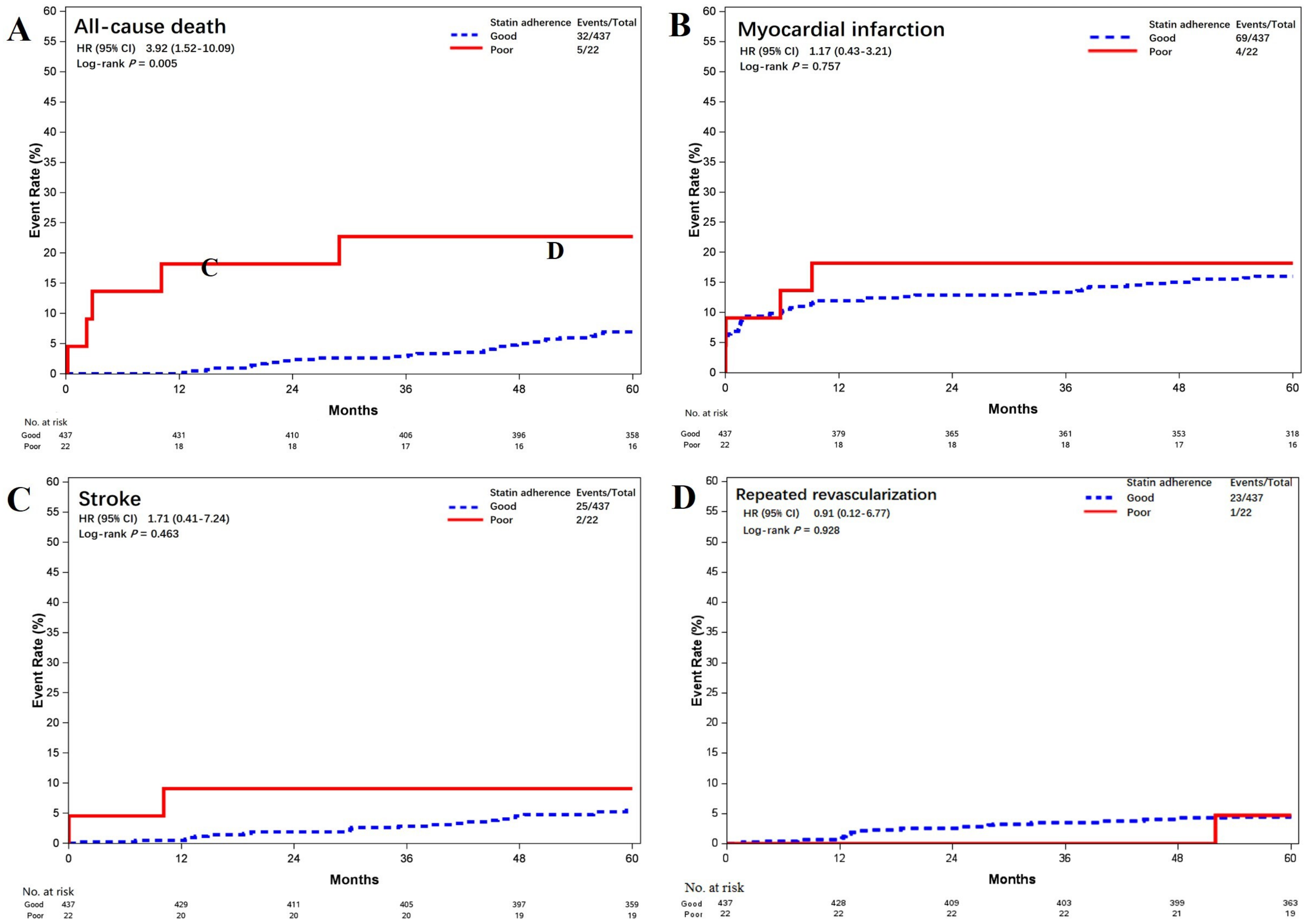

Kaplan-Meier estimates for freedom from **(A)** all-cause death, **(B)** myocardial infarction, **(C)** stroke, and **(D)** repeated revascularization.
