# Supplementary material for: Impact of low-density lipoprotein cholesterol and lipoprotein(a) on mid-term clinical outcomes following coronary artery bypass grafting: A secondary analysis of the DACAB trial
Source: Front Cardiovasc Med. 2023 Mar 24;10:1103681. doi: 10.3389/fcvm.2023.1103681 (PMC10080087; doi:10.3389/fcvm.2023.1103681)
Supplement: Supplementary file 2 [file Table1.pdf]

**Table E1 Baseline characteristics of the study population stratified by 1-year statin adherence**			
**Characteristics**	**Good statin adherence (n=437)**	**Poor statin adherence (n=22)**	***p* value**
Age, years, mean (SD)	63.1 (8.1)	65.1 (9.2)	0.262
Male, n (%)	357 (81.7)	17 (77.3)	0.577
**Clinical status, n (%)**			0.862
CCS	151 (34.6)	8 (36.4)	
ACS	286 (65.5)	14 (63.6)	
**Medical history, n (%)**			
Myocardial infarction	136 (31.1)	7 (31.8)	0.945
Hypertension	328 (75.1)	20 (90.9)	0.090
Diabetes mellitus	199 (45.5)	12 (54.6)	0.408
Peripheral artery disease	73 (16.7)	6 (27.3)	0.241
Stroke	47 (10.8)	5 (22.7)	0.090
Chronic kidney disease	6 (1.4)	2 (9.1)	0.052
**Cigarettes smoker, n (%)**	212 (48.5)	13 (59.1)	0.333
**NYHA, n (%)**			0.118
I+II	267 (61.1)	11 (50.0)	
III+IV	170 (36.6)	11 (50.0)	
**LVEF, n (%)**			0.773
<40%	5 (1.1)	0	
40%-49%	40 (9.2)	1 (4.5)	
≥50%	392 (89.7)	21 (95.5)	
**SYNTAX score, n (%)**			0.908
Low (0-22)	64 (14.6)	2 (9.1)	
Medium (23-32)	241 (55.1)	13 (59.1)	
High (≥33)	132 (30.2)	7 (31.8)	
**Euro Score, n (%)**			0.242
Low (0-2)	176 (40.3)	7 (31.8)	
Medium (3-5)	201 (46.0)	9 (40.9)	
High (≥6)	60 (13.7)	6 (27.3)	
**Baseline LDL-C levels, mmol/L, n (%)**			0.486
<1.8	127 (29.1)	8 (36.4)	
1.8- <2.6	196 (44.9)	7 (31.8)	
≥2.6	114 (24.1)	7 (31.8)	
**Postoperative LDL-C levels, mmol/L, n(%)**			0.050
<1.8	124 (28.4)	5 (22.7)	
1.8- <2.6	181 (41.4)	5 (22.7)	
≥2.6	132 (30.2)	12 (54.6)	
**Lp(a) levels, mg/dL, n (%)**			0.337
<30	315 (72.1)	13 (59.1)	
≥30	122 (27.9)	9 (40.9)	
**Antiplatelet therapy, n (%)**			0.045
Aspirin	147 (33.6)	4 (18.2)	
Aspirin+Ticagrelor	146 (33.4)	13 (59.1)	
Ticagrelor	144 (33.0)	5 (22.7)	
**Medication at discharge, n (%)**			
Beta-blocker	394 (90.2)	22 (100.0)	0.242
ACEI/ARB	260 (59.5)	14 (63.6)	0.767
Statin	416 (95.2)	21 (95.5)	0.533
**Surgical characteristics, n (%)**			
On-pump	99 (22.7)	4 (18.2)	0.796
IMA user	370 (84.7)	13 (59.1)	0.005
